# Towards the Automated Analysis and Database Development of Defibrillator Data from Cardiac Arrest

**DOI:** 10.1155/2014/276965

**Published:** 2014-01-12

**Authors:** Trygve Eftestøl, Lawrence D. Sherman

**Affiliations:** ^1^Department of Electrical and Computer Engineering, Faculty of Science and Technology, University of Stavanger, 4036 Stavanger, Norway; ^2^Department of Medicine, University of Washington, 999 3rd Avenue, Suite 700, Seattle, WA 98104, USA; ^3^Department of Bioengineering, University of Washington, 999 3rd Avenue, Suite 700, Seattle, WA 98104, USA

## Abstract

*Background.* During resuscitation of cardiac arrest victims a variety of information in electronic format is recorded as part of the documentation of the patient care contact and in order to be provided for case review for quality improvement. Such review requires considerable effort and resources. There is also the problem of interobserver effects. *Objective.* We show that it is possible to efficiently analyze resuscitation episodes automatically using a minimal set of the available information. *Methods and Results.* A minimal set of variables is defined which describe therapeutic events (compression sequences and defibrillations) and corresponding patient response events (annotated rhythm transitions). From this a state sequence representation of the resuscitation episode is constructed and an algorithm is developed for reasoning with this representation and extract review variables automatically. As a case study, the method is applied to the data abstraction process used in the King County EMS. The automatically generated variables are compared to the original ones with accuracies ≥90% for 18 variables and ≥85% for the remaining four variables. *Conclusions.* It is possible to use the information present in the CPR process data recorded by the AED along with rhythm and chest compression annotations to automate the episode review.

## 1. Introduction

During resuscitation of cardiac arrest victims automated external defibrillators (AEDs) record a variety of information in electronic format. In many emergency medical service (EMS) systems this electronic information is downloaded to a computer system as part of the documentation of the patient care contact and in order to be provided for review of the case for quality improvement activities. The electronic information will then be available as digital files which include physiological signals and also operational data related to the defibrillator (energy delivered, mode: automatic or manual, impedance, time of each event, etc.) logged from the defibrillator. Data related to operation of the defibrillator we denote as “CPR process data.” This data may be organized and stored in a registry of the cardiac arrest cases. This registry may then serve as a database that may be used in studies of resuscitation strategies directed at improving survival from cardiac arrest. The collected physiological data includes the electrocardiogram recording the cardiac activity of the patient and depending on the recording features of the device, the impedance between electrodes, the acceleration and force of chest compressions, end tidal CO_2_, blood pressure, and possibly other biometric measures. The CPR process data defined above also carries essential information about critical time points such as the exact time the device is turned on, the results of each shock advisory analysis, and the precise time of defibrillation shocks. In addition to the CPR process data that the device may produce, there are various written or electronically generated reports documenting the resuscitation episode along with clinical and demographic information. These reports are filed by dispatching centers and by the EMS responders during and following the resuscitation. In many systems an audio recording is made allowing a listener to review the course of resuscitation to supplement the ECG presentation and written reports.

It is our belief that the integration of CPR process data combined with an automatic analysis of the physiologic signals would make it possible to objectively and efficiently analyze resuscitation episodes in an objective reproducible format. Such analysis is important as it provides the means for analyzing and archiving parameters describing the quality of cardiopulmonary resuscitation (CPR). A simple example is the ratio of hands off intervals (HOI) during therapy. In several studies both ECG and chest compression tracings have been reviewed to accurately identify all such HOI. These studies have shown that, despite the subjective impression by rescuers that CPR delivery was adequate, in fact the HOI duration exceeded the recommendations given in the resuscitation guidelines [1, 2]. These findings had a significant impact on the 2005 guidelines revision [3]. As a result, an increased focus and attention to continuous uninterrupted chest compressions has had a positive effect on survival rates as reported in several studies [4–7]. For these studies to give significant results, quite a large number of resuscitation episodes were collected and reviewed. This involved considerable effort for the reviewers and careful definitions of the parameters to be recorded in order to make the resulting analysis objective and relevant. Interobserver variation is a significant confounding factor in these studies. The interpretation often involves determining the cardiac rhythm and the transitions between rhythms both with and without the presence of chest compressions. These rhythm and chest compression annotations involve interpretation of the ECG for rhythm assessment and of the compression signals for identifying chest compression sequences.

The present study is undertaken in order to determine whether it is feasible to automate the process of data analysis and extraction of the clinically relevant features. In particular we seek to demonstrate that information which is currently collected by manual review of cases of ventricular fibrillation cardiac arrest involving many hours of review can be replicated using an automated extraction technique. This will be done through the following three steps: (1) The concept of a minimal information set defined by important events during the resuscitation is proposed. (2) From the minimal information set a state sequence model is constructed. (3) Algorithms are designed to reason over the state sequence model to automatically replicate the manual interpretation of CPR process data.

## 2. Methods

There are several layers of information involved in the interpretation of a resuscitation episode. Some of the clinical variables are derived directly from the CPR process data and annotations of rhythm and therapeutic events and are therefore fundamental or primary. Other variables, the secondary variables, can be inferred or calculated from the primary variables. From these primary information variables we furthermore propose a state sequence model from which it will be possible to design algorithms to perform the reasoning to infer the secondary variables.

### 2.1. Defining the Primary Information Objects

In developing the automation of such a process it was necessary to consider the type of information to be retrieved. Some objects of information are more fundamental than others. One may distinguish a hierarchy of these objects as primary and secondary in the sense that the secondary objects may be determined from the information present in the primary objects. Our hypothesis is that the secondary objects of information can be derived automatically from the primary objects by designing an algorithm that reasons on the primary objects to produce the secondary objects. It is our hypothesis that these primary objects include a subset of the elements in the AED event record and of the annotations of rhythm transitions and of the start and stop times of the chest compression sequences.

To formalise this concept, we associate these primary information objects to categories of important events during a resuscitation episode. A resuscitation episode can be described as an episode starting at time *t*_*s*_ and ending at *t*_*e*_. Throughout the episode, there are important events *e*_*i*_ that can be associated with a given time *t*_*i*_. In our model of resuscitation, we define two important categories of events: *therapeutic *and *rhythmic* events. (We will also refer to rhythmic events and states as response events and states.) The therapeutic events are limited to the set *T*_*e*_ = {c1, c2, d1, d2} marking the start and end of a compression sequence (c1 and c2) and start and end of a defibrillation (d1 and d2).

The rhythmic events represent rhythm transitions which we limit to the set *R*_*e*_ = {vf, vt, as, pe, pr} marking transitions into ventricular fibrillation (vf), ventricular tachycardia (vt), asystole (as), pulseless electrical activity (pe), and pulse giving rhythm (pr).

**Figure 1:**
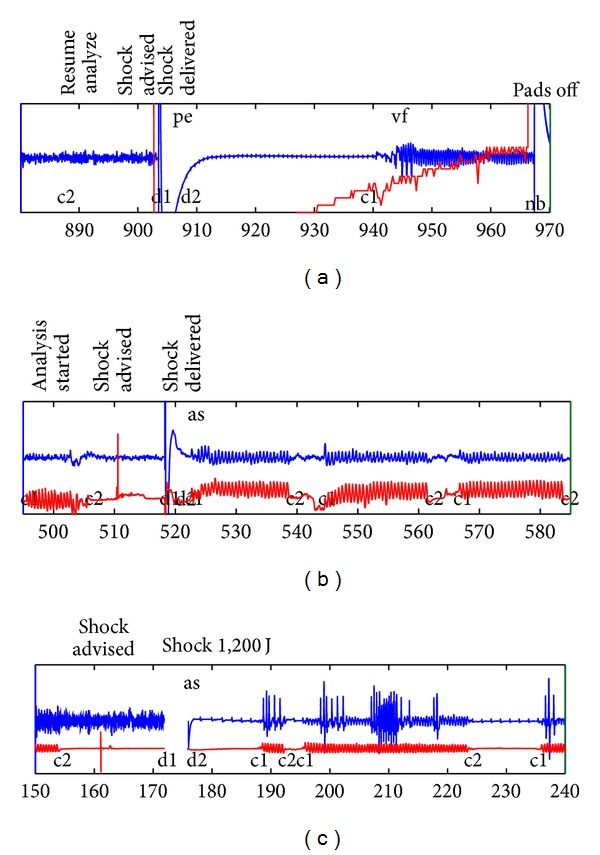
Signals and data recorded by three different automated external defibrillators: (a) Philips Forerunner 2, (b) Philips MRx, and (c) Physiocontrol Lifepak 12. The blue and red tracings show the electrocardiogram and thoracic impedance, respectively. Examples of information recorded in the defibrillator's electronic log are shown above each plot window. Annotations of rhythm transitions and therapeutic events are shown at the top and bottom inside each plot window.

Examples of both types of annotations are shown at the top and bottom inside each plot window of the tracings of the AED signals in Figure 1 where CPR process data and annotations are shown for three different types of AEDs.

The first step in the automated review process will be to collect these events or primary information objects which we denote as PIOs from the manually recorded data and from the defibrillator. These PIOs will be processed to construct the state sequence which we denote as the “Representation of the Resuscitation Event” (RORE). The RORE (to be discussed in more detail below) is then input to the reasoning algorithm which produces the secondary information objects (denoted as SIO). The derived database can then be compared to the original database to determine the accuracy and validity of using only the PIOs to determine and define all of the information present in the database. This serves the twofold purpose of defining the minimal dataset (PIOs) which needs to be collected by an automatic algorithm designed for this purpose and also tests the ability of the RORE created only from the PIO to serve as the sole source for an accurate clinical database to be used in resuscitation research and quality improvement activities.

### 2.2. Using Primary Information Objects to Create Representations of Resuscitation Episodes

In a previous article Eftestøl et al. presented a conceptual framework for representing the data from resuscitation episodes [8]. This representation is essentially a standardized data format developed to describe a resuscitation episode. Briefly, the RORE involves two aspects that are separately modeled, the therapy domain state sequence and the response (or rhythmic) domain state sequence. Generally, we denote a resuscitation episode as a sequence of changing states. The individual states are defined initially within either the therapy or the response domain and these two aspects are put together in a combined sequence to concisely describe the resuscitation. A change in either domain constitutes a change in the state of the combined episode representation. The transitions between the states in each domain are represented as delimited time intervals, where the start and end time for each interval is given for that state. The start and end times therefore indicate the time of transition into and out of the represented state. The time of transition out of a specific state corresponds to the time of transition into the next state. For the response domain, the states are the various cardiac rhythms that occur throughout a resuscitation episode. In the therapy domain, the states are the interventional treatments given to the patient: in this study these were the chest compression sequences, hands off intervals, and defibrillation shocks. These two sequences or representations and the combination of these two domains constitutes the RORE. 

*A Formalised Description of the Concept in the Context of the PIO Events.* To be able to design algorithms that can reason on the information we need a model that captures the relationship between the elements both in terms of the course of time and type of event.

To each type of event *e*_*i*_, there is the time point *t*_*i*_ describing the transition or onset time. One can say that the event marks a change of state, the state *E*_*i*_ determined by the type of event at time *t*_*i*_. The state is unchanged until the next event *e*_*i*+1_ marks the transition into the next state *E*_*i*+1_.

To each state, we define the corresponding time interval, *T*_*i*_ = [*t*_*i*_, *t*_*i*+1_]. Thus, the course of events during a resuscitation episode will be defined as a continuous sequence of states *S* = {(*T*_1_, *E*_1_), (*T*_2_, *E*_2_),…, (*T*_*N*_, *E*_*N*_)} where the time intervals are ordered according to time since start of episode, *t*_*s*_.

We define three sets of states. The first two sets are related to the therapeutic and rhythmic events. The therapeutic states are limited to the set *S*_*e*_ = {C, H, D} marking the compression sequences (C), the hands-off intervals (H), and the defibrillations (D). The rhythmic states are limited to the set *S*_*r*_ = {VF, VT, AS, PE, PR} which represents ongoing rhythms the start and end of which are defined by the corresponding transition events. VF is the state ongoing in the time interval the start of which is marked by the transition event vf and ended by one of the other transition events in *R*_*e*_. The relationship between the other rhythmic events as, vt, pe, and pr and states AS, VT, PE, and PR is similar. The third set is constructed from the combination of the therapeutic and rhythmic states in the time interval where the two types of states are unchanged. If the rhythmic state sequence is

(1) $$\begin{array}{c} S_{R}=\left\{\left(\left[t_{0},t_{3}\right],E_{R1}\right),\left(\left[t_{3},t_{4}\right],E_{R2}\right)\right\} \end{array}$$

and the corresponding therapeutic state sequence is

(2) ST={([t0,t1],ET1),([t1,t2],ET2),  ([t2,t4],ET3)},

the combined state sequence will be

(3) SC={([t0,t1],ET1ER1),([t1,t2],ET2ER1),  ([t2,t3],ET3ER1),([t3,t4],ET3ER2)}.

Note how the state labels from *S*_*R*_ and *S*_*C*_ are concatenated.

For the tracing in Figure 1(a), the three state sequences representing the part of the resuscitation episode that is shown will be as follows:

(4) ST={([740.8,805.2],H),([805.2,887.2],C),  ([887.2,903.3],H),([903.3,908.3],D),  ([908.3,938.8],H),([938.8,1062.0],C)},SR={([793.6,908.3],VF),([908.3,944.0],PE),  ([944.0,1062.0],VF)},

and the combined sequence will be

(5) SC={([793.6,805.2],HVF),([805.2,887.2],CVF),  ([887.2,903.3],HVF),([903.3,908.3],DVF),  ([908.3,938.8],HPE),  ([938.8,944.0],CPE),([944.0,1062.0],CVF)}.

### 2.3. Designing Algorithms Reasoning on the RORE

The RORE is well suited for designing reasoning algorithms which aims to mimic the interpretation a clinician will do. The basic principle is that the algorithms can identify time intervals in the state sequences fulfilling criteria expressed by the state sequence labels. For example, in the current study, the RORE was implemented in MATLAB where the state sequences can be realised as a list with the sequence labels. The time intervals are placed in corresponding lists so that, if a specific state symbol is found in position *i* in the list of state sequence labels, the corresponding time interval can be found in position *i* in the list of state sequence time intervals. The complete episode from which the tracing in Figure 1(a) originates is represented by the therapy sequence *S*_*T*_ = {H, D, H, C, H, D, H, C, H, C, H, C, H, C, H, C}, the rhythm sequence *S*_*R*_ = {VF, PE, VF, PE, VF, PE, VT, VF, PE, VF, UN}, and the combined sequence *S*_*C*_ = {HVF, DVF, HPE, CPE, CVF, HVF, DVF, HPE, CPE, CVF, CPE, HPE, CPE, HPE, HVT, HVF,CVF, HVF, CVF, HPE, CPE, CVF, CUN}. (Notice the use of UN for unknown state.) The framework is quite flexible where the RORE serves as the vocabulary on which it is possible to reason to derive the SIOs. For example, the initial rhythm can be determined by retrieving the first element from SR. The time of the defibrillations can be determined by searching ST for occurrences of D and retrieving the corresponding time intervals.

As we will see later, the review will be focused on the pre- and postshock periods of each shock. This can be done by repeating the analysis for each shock, wherein the state sequences between the current and previous shock (or beginning of episode) are extracted to represent the preshock period. The states between the current and next shock (or end of episode) are extracted to represent the postshock period. Subsequently, the start of the first compression sequence and the end of the last compression sequence can be found by searching the preshock therapy sequence for the first and last occurrences of C. The duration of the preshock compression sequence can furthermore be by subtracting the start time for the time interval of the first C from the end time of the last C.

We will use this kind of reasoning to illustrate how this methodology can be used in the following case study.

## 3. A Case Study

The extraction from the King County database utilized in the current study will act as a model for the design of automatic data collection algorithms which is the goal of this study. By using this manually acquired data, a particular representation of the cardiac arrest for each subject will be developed which will contain the candidate set of variables to exactly describe and document the resuscitation episode. These automatically derived variables will be a replica of the original variables and the two data sets will be compared for evaluation.

### 3.1. Current State of the Art

The EMS division of King County has registered all sudden cardiac arrests treated in a large metropolitan area surrounding Seattle, WA, since 1976 [6, 9, 10]. This database has been used in several retrospective studies, where the study objectives have been diverse. Specific examples include the recording of Utstein elements to investigate long term survival among resuscitated patients [9], the effect of time to EMS arrival on survival [10], and the effect of resuscitation algorithm changes on survival [11], and the application of public access defibrillation affects EMS therapy [12]. In the scope of the present investigation, studies using the information derived from interpretation of the CPR in association with the analysis of the physiologic signal are of particular relevance. For example, in one study the effect of a change in the cardiac arrest protocol introduced to decrease the hands off interval (HOI) associated with shock delivery was assessed [6]. The effect of the protocol change was evaluated by analyzing time intervals before and after shocks. In another study designed to develop a method to predict the outcome of defibrillation, ECG waveforms prior to the first shock were extracted for analysis along with information regarding whether ROSC occurred following the defibrillatory shock [13]. For all of these studies, the abstraction of information was carried out by following a carefully scripted case review routine. This abstraction process includes review of the EMS medical incident report forms using specifically designed forms based on Utstein variables, review of electronic recordings from the AEDs, again using well-defined criteria, and using predefined time points for rhythm assessment. In addition there was direct audio review of dispatch recordings for each case. These records are reviewed to determine various aspects of therapy, such as duration and frequency of chest compressions, number and timing of defibrillations, response to shocks, and the presence and duration of HOI. In King County the review process is clearly defined in a data dictionary where each variable generated from the review is listed with an explanatory description and the possible values it can have. After the review, the variables are stored in an Access database (Microsoft Corporation). The review itself is conducted by dedicated staff members who receive extensive training in abstraction techniques prior to performing independent reviews. All cases are abstracted by a minimum of two reviewers. All cases with conflicts are adjudicated by a supervising physician expert in ECG analysis.

### 3.2. Overview of Data Collection

**Figure 2:**
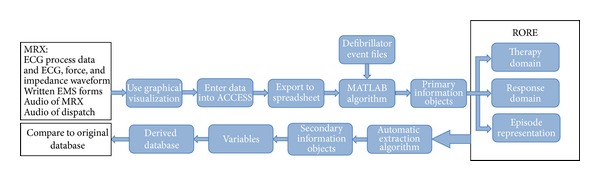
The process of deriving the variables from the manual review; the generation of the representations used for the further algorithmic reasoning for deriving the variables automatically.

The total data set consisted of a convenience sample of 75 cardiac arrests from the King County registry which were completely deidentified using custom software written for this purpose. The study was approved by the IRB of the University of Washington. The study was divided into two phases. In the development phase, 20 cases were randomly selected and used to create the algorithm which utilized the two sources of clinical information available. The algorithm used both the raw data from the original database which had been placed in Excel formatted files and the CPR process data (from the defibrillator downloads of the defibrillator data files) to create the summary of the case termed the “Representation Of the Resuscitation Episode” (RORE). Then this process was reversed so that the RORE was used to recreate a second database whose purpose was to determine if the original data could be accurately abstracted from the RORE to recreate the clinical record in an automatic algorithmically driven manner. After the method had been adjusted to perform well on the development set data, its accuracy was then tested on a validation set of the remaining 55 cases from the database (Figure 2).

### 3.3. Overview of the Variables to be Replicated

To begin the process, data fields of interest were extracted from storage in the King County EMS repository which is an Access database by being exported to an Excel spreadsheet. The patient population was restricted to those treated with MRx AED devices (Philips Inc.) which uses a small “puck” placed under the rescuers hands during chest compressions to very accurately record the force and acceleration used in chest compressions. The CPR process data consisted of the electronic files stored by the MRx during each resuscitation episode. The electronic data were downloaded to computer archives immediately after the episode. The written report of the patient care encounter as filed by the EMS crew following the resuscitation was also used in the manual abstraction process.

There are several types of variables in the database. Variables describing the defibrillation shocks, chest compressions, and patient response were included. Each of these general categories of variables contain subsections which detail the time of each event, the operation of the device (i.e., Joules delivered with each shock, impedance at each shock, etc.), and variables describing transitions in rhythm related to the patient's response after each shock. Each row in the Excel spreadsheet stores the variables related to one specific shock and includes the following.

*(1) Time Events.* Each time event is given by three variables, hour, minute, and second of the day. For example, “ECG start time” corresponding to the AED power on time is registered in the three variables: ecghr, ecgmn, and ecgsc. Each of these is coded numerically: 0–23 for hour, 0–59 for minutes and seconds. We will refer to these collectively as ecgtime (hr:mn:sc) denoted by the variable name “ecgtm.” The other time data points reported for a shock include the events describing therapy prior to and including the shock: “First compression time,” “Last compression time,” and “Shock time” (variable names “fctm,” “lctm,” and “shktm”). The time events following a shock describe the patient's response and give the time points for transitions to specific rhythms after each shock is given: “VF onset time” and “ROSC time” (return of spontaneous circulation) (variable names “vfonsettm” and “rsctm”).  Table 1 shows a more detailed view of the time event variables.

*(2) Device Operation and Therapy.* “Shock number,” “Number of shock sequences recorded,” and “Number of shocks in sequence” record the shock sequence number, the number of electrical shocks delivered to the patient, and the number of “stacked” shocks without intervening CPR (variable names “shkn,” “ssrecord,” and “shks”). The AED operating mode, either “manual” or “advisory,” was coded in the variable “mode.” EMS provision of CPR was coded in the variable “CPR.” The energy and impedance of the shock are given by the variables “enrgy” and “imp,” respectively.  Table 2 shows a more detailed view of the device operation and therapy variables.

*(3) Patient's Response Following a Shock.* For the initial rhythm, the variable “init_rhy” describing the rhythm at the start of the ECG recording was captured. The rhythms at 10, 30, 60, and 120 seconds after the shock were recorded in the variables “r10,” “r30,” “r60,” and “r120.” These time points were originally developed in an effort to determine the outcome of the defibrillatory shock as precisely as possible. It was felt that the first 2 minutes after shock were most important in determining if a shock had been successful in producing an organized and possibly perfusing rhythm and that, by using discrete, well-defined points, the rhythm changes could be determined in a time window of relevance to evaluating the effect of resuscitation therapies. In addition, the variable “orgpr” is used to describe whether there was an organized rhythm at any time during the interval before the next shock (or the end of the recording if no more shocks were given). In a similar manner “vfpr” indicates whether there is a recurrence of VF prior to the next shock.  Table 3 shows a more detailed view of the patient's response variables.

*Primary and Secondary Information Objects.* Table 4 provides an overview of the primary information objects that we use to construct the RORE. Considering the variables registered during the manual review, some of these basically reflect the same information as the primary information objects, while the remaining variables correspond to secondary information objects.  Table 5 provides an overview of all the variables handled in this study. Variables are categorized as describing time events, patient response, and device operations and therapy and primary and secondary information objects are indicated by the acronyms PIO and SIO, respectively.

### 3.4. Generating the Representations

**Figure 3:**
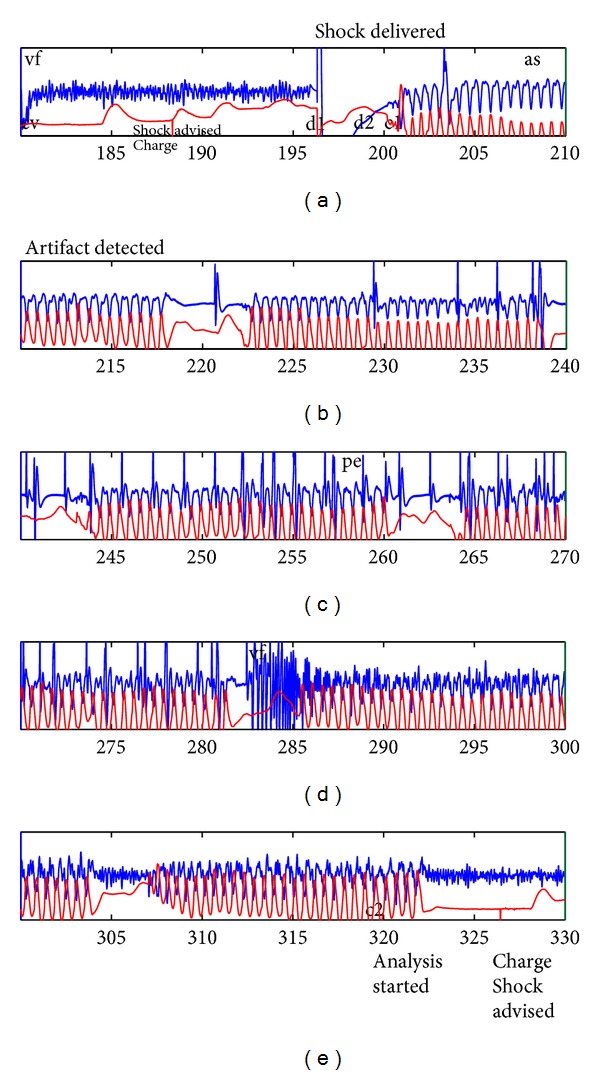
Case recording from MRX defibrillator. The blue tracing is the ECG. Impedance tracing is shown in red. The defibrillator log events (shock advised, charge, shock delivered, analysis started, and artifact detected), the rhythm transitions (vf: ventricular fibrillation, as: asystole, and pe: pulseless electrical activity), and annotations for start and end of compressions (c1 and c2) and defibrillations (d1 and d2) are shown. Refer to Figure 4 for the corresponding RORE representation.

To generate the RORE, the information recorded by the defibrillator and contained in the CPR process data on the device is used directly for the time and shock data: that is, the time the device is turned on, the exact time of each shock, number of joules delivered, impedance at time of shock, and so forth are obtained directly from the device event files. In addition, the information for rhythms and chest compressions extracted by manual review of the ECG using Event Review 3.1 is then recorded in the ACCESS database and subsequently placed in the Excel spreadsheet (Figure 2). Several PIOs are extracted from both the written EMS reports and from the manual review. We have observed and wish to stress that, in order to accurately describe the rhythm data, the only data points required are the times of rhythm transitions between different rhythm types. This decreases the amount of information stored in the RORE substantially. This is clearly illustrated in the following example (see Figure 3).

**Figure 4:**
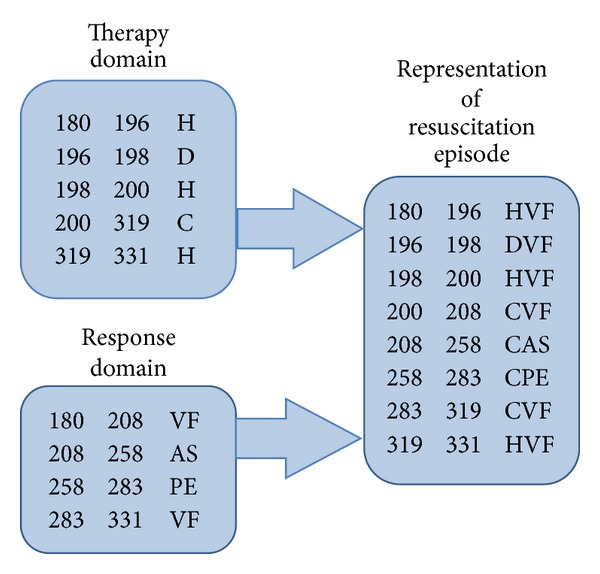
The representation of the resuscitation episode (RORE) including the therapy domain representation, the response domain representation, and the episode representation (for detailed explanation see Appendix C). UN = unknown, C = compressions, H = hands off chest, D = shock, CVF = compressions during VF, CAS = compressions during asystole, DVF = defibrillation for VF, PE = pulseless electrical activity, and PR = pulsatile rhythm.

In this resuscitation we are viewing the ECG (blue) and the thoracic impedance (red). The ECG represents the cardiac activity while the impedance indicates the chest compressions. The time interval shown here is from 180 seconds to 330 seconds. This shows a section of VF beginning at 180 seconds with no CPR being performed and during which analysis of the ECG has recommended a shock. The shock is delivered at 198 seconds (d1) and CPR begins at 201 seconds. The CPR artifact shows a possible QRS complex at 204 seconds and a definite QRS at 221 seconds and again at 229 seconds. In the database used for this study the rhythm was recorded at predetermined time points (10, 30, 60, and 120 seconds). An organized rhythm was defined as being at least 2 beats within the 5 seconds before and after the time point. Since the 30-second time point is 228 seconds this does not qualify as an organized rhythm yet. At 60 seconds (258 sec.) an organized rhythm is present without a pulse being detected. VF then recurs at 282 seconds and persists with CPR artifact until the end of the trace. One can easily appreciate that the description of this 150-second period of resuscitation is complete but quite lengthy. To condense the description for the KCEMS database we attempted to reduce the record by focusing on specific time points immediately after the shock. This is recorded in the database as described above with the rhythm at each time point recorded. Because discrete predetermined time points are used there is an inherent inaccuracy in the record. The rhythm transition points are only estimated by the time points used and then only if the transition occurs within the first two minutes after shock. In contrast, for the PIO based data recorded in the response domain, only the transitions in rhythm are recorded and these would ideally be recorded at the precise time of occurrence. The episode in the lengthy description above is shown in RORE format in Figure 4. For the 180 to 330 time interval there are 5 lines of notation in the therapy domain, 4 lines in the response domain, and 8 lines in the overall episode representation (Figure 4 outlined portion). Ideally, the rhythm transitions would be determined exactly by direct manual review or by an automatic computer algorithm with an overread by a reviewer. This would provide the exact times of transition. In this study, to determine “proof of concept,” the KCEMS database was used to derive the time points of transitions, while recognizing that they would be only estimates of these transitions. Using the KCEMS database the response domain is formed as follows. The events in the database are recorded in seconds so that the initial rhythm of VF is noted to begin at 46 seconds after the defibrillator is turned on at which time the leads are connected. The next rhythm transition is to asystole at 208 seconds (r10, 10 seconds after the shock) followed by transition to an organized rhythm without a pulse at 258 seconds (r60, 60 seconds after the shock) and a return to VF at 283 seconds. Note that the reoccurrence of VF was identified as accurately as possible by the reviewers while the organized rhythms were recorded only at the 4 time points. The response domain for these events is shown in seconds as “46–208: VF” and then “208–258: AS” to indicate asystole as the rhythm at 10 seconds after the shock. Then there is a transition to an organized rhythm without a pulse: “258–283: PE” and another transition when VF recurs: “283–343: VF.” This method produces a succinct record of rhythms as shown in Figure 4.

The therapy domain states are presented in a similar manner. The chest compression times are read directly from the variables for first and last compression times prior to the each of the shocks present in the ACCESS database. All other information is read from the CPR process data as recorded in the MRX defibrillator. For example, in the defibrillator event log all events are listed along with the time in milliseconds. The time for each shock can be found by doing a search of the MRx recorded data for the text string “shock delivered” indicating the shock events. Once found, the corresponding time is given in milliseconds and is converted to seconds. This is illustrated in Figure 3 in which the defibrillator log events such as “shock delivered” can be seen outside the trace boxes. In this figure the rhythms noted in the KCEMS database have been inserted as rhythm annotations such as “vf” and “as” and are seen in the upper portion just inside the boxes. The therapy related annotations shown at the bottom inside of the boxes (“c1”/“c2” and “d1”/“d2”) are the start and end of compression sequences and defibrillation events respectively). The therapy domain representation was created from the KCEMS database in a manner similar to that described above for the response domain. For the 180–330-second period shown in Figure 3 there is a hands off interval from the start at 180 seconds to the time of the shock at 196 seconds. After the shock follows a brief hands off interval continuing until compressions start at 200 seconds which continues with brief pauses for ventilations until chest compressions are halted at 322 seconds. Lines 3–7 in the therapy domain (Figure 4) represent this period of the resuscitation. The RORE is easily constructed by combining the therapy and response representations and gives a comprehensive picture of the relationship between the provided therapy and the patient response to the treatment. In the following, the therapy domain, response domain, and the RORE make up the completed representation using the PIOs as described above.

### 3.5. Reasoning from the Representation Back to Create the Derived Data

The working principles of the reasoning algorithms are described in the following. For increased readability a prose form has been chosen rather than using a pseudocode description. All the algorithms have been implemented and run in MATLAB.

Before beginning to work back from the representations to the database it is necessary to establish the precise absolute time for the resuscitation. The KCEMS database uses the absolute times at the data points as extracted by manual review of the record. The times in the defibrillator log file and subsequently the representation are relative or elapsed times. In order to be able to calculate back to the absolute time used in the manual registration it was necessary to use the time as recorded in the stored defibrillator files at the time the device was turned on as the reference time for all events. Doing this involved calculations to convert the start time from the conventional time in year/month/day/hour/minute/second format to a “serial date number” time (the number of days from January 1, 0000) used by the algorithm. These times are then converted to elapsed times for the representations. The elapsed times are then converted back to absolute times via the “serial date number” time for comparison to the original times in the database. The comparison of the times from the representations to the database can therefore be viewed as a comparison of the accuracy of the manual method of extraction by the reviewer to the automated method based on the defibrillator's internal files. In the automated method the exact time the defibrillator is turned on is used as the basis for developing the times of shocks and other events. The assumption is made that the defibrillator has been correctly synchronized with the local regional time. (See Appendix A.1 for details.)

The first primary information objects to be established and compared are the defibrillation shocks. Using the therapy domain representation, each shock in the therapy domain is identified and its position noted; the absolute time is calculated. Then the preshock and postshock periods can be identified for each shock. A preshock period is the time interval between the current shock and the previous shock and the postshock period the interval between the current shock and the next shock. If the current shock is the first shock the previous shock is replaced by the beginning of episode (BOE) marker. Likewise if it is the last shock of the recording the “next shock” is replaced by the end of episode (EOE) marker in the therapy domain. To determine the “First compression time,” the preshock period of the current shock as recorded in the therapy domain is used as the time interval within which to search for the first occurrence of the symbol used to identify compressions, “C.” Likewise, “Last compression time” is determined by searching the recorded preshock interval for the last occurrence of “H” which signifies a change in state from CPR to “hands off time” prior to the defibrillatory shock “D.” The times for these PIOs are then converted to absolute times.

The PIOs for the rhythm domain are handled in a similar manner. Here the PIOs represent the rhythm domain states and are VF (ventricular fibrillation), AS (asystole), PE (pulseless electrical activity), PR (pulsatile rhythm), and UN (unknown). These states are represented in the RORE. Custom software was programmed to identify the preshock and postshock periods of each shock in the RORE and then to search these intervals for the first occurrence of the PIO of interest (VF onset or ROSC onset). The time associated with this event is then converted as noted above from seconds to a computer time stamp known as SDN_time (see Appendix A.1 for details) to hr:mn:sc format and is then compared to the original KCEMS database.

The next step in the conversion process from the RORE to the derived database is to derive the secondary information objects (SIOs; Table 5) from the PIOs. Determination of the Ecg_start_time and other time variables has been described with the time conversion process. The rhythm variables are handled by assuming that once a rhythm is present that it remains in that state until the notation in the RORE indicates a change in the rhythm state. The algorithm recreates the rhythm at a specific time point simply by identifying the time interval in the RORE containing this time point and noting the corresponding rhythm symbol in the RORE occurring immediately before this (for detail: Appendix A.2). To determine if there are occurrences of VF or organized rhythms before the next shock, the postshock interval is searched for occurrences of the types of rhythms in question and the results allocated to the variables “vfpr” and “orgpr.”

The device operation variables are also directly related to the defibrillatory shocks. The number of each shock, “shkn,” is determined by sorting the shocks in ascending order according to the sample numbers and assigning to each shock the number corresponding to its position in the ordered sequence. The number of shocks without intervening CPR is determined by initializing a counter to one. For each shock, the preshock interval is searched for compression events. If none are found, the counter is incremented. The counter value is recorded in the variable “shks.” The variable, “ssrec,” which gives the number of recorded shocks is determined as the length of the ordered sequence of shocks. In the database, the “mode” variable indicating manual or AED mode is based on the clinical impression of the reviewer rather than on the CPR process data from the defibrillator log files. In the algorithm implementation the “modeSwitchMonitor” and “modeSwitchAED” in the defibrillator log file is used directly. Once identified for each shock the mode is compared to the database in accordance with the latest such entry prior to the time of shock. The energy and impedance variables are also manually estimated in the database by the reviewer. In contrast, in the algorithm these were read directly from the defibrillator log file in the information provided with each shock. These values are then compared.

### 3.6. Comparing the Original and Replicated Databases

For result evaluation, each of the automatically generated SIO variable values based on the RORE representation are compared to the corresponding manually registered values read into MATLAB from the Excel spreadsheet. These comparisons were coded according to being correct, wrong, or missing. These three categories are given the numeric codes 1, 2, and 3, respectively. The comparisons involve computing the value difference and comparing this to the specified value ranges for each of the three categories. As all values are integers, the deviations will also be integers. The value ranges are provided in the next section.

When comparing recorded event times, a deviation of 1 second or less was defined as correct, those larger than this were defined as wrong, and deviations due to the codes for persistent VF, no CPR data, and no data available were defined as “missing”(see Appendices B.1 and B.2 for details).

After tuning the system on the first twenty, it was run on the 55 episodes that had not been interpreted by the system previously. One episode was excluded as the registration was incomplete (shocks 2 and 3 missing). Error rates and a detailed analysis of the etiology of each error were performed.

## 4. Results

**Figure 5:**
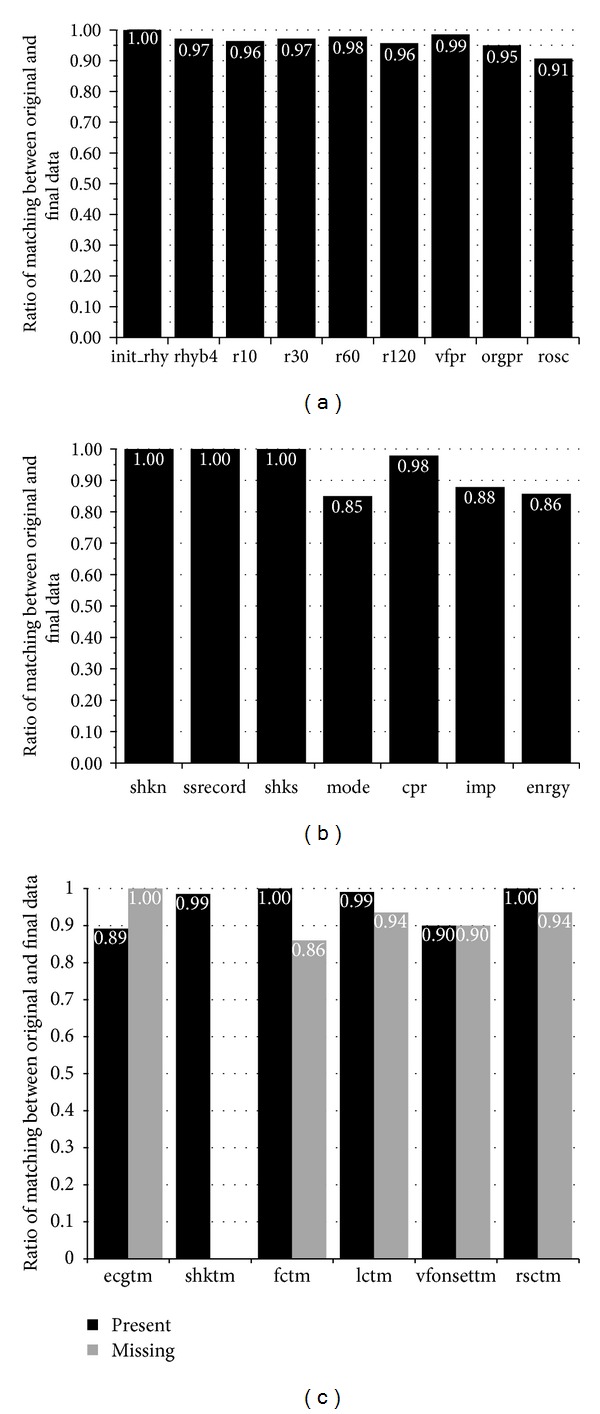
Summary of the match rates comparing the manually and automatically derived variable values. (a) shows match rates for rhythms at the preselected times from the database. (b) shows data regarding the defibrillator logs of shock data. (c) shows the times for ECG start, shock times, first compression and last compression times, VF onset times, and time of return of spontaneous circulation. All results are given in percentages of the ratio: correct/(correct + error). For the time variables, the lower row shows the ratio of automatically generated codes for missing values matching the manually given missing codes (gray bars).

Error rates are shown in Figure 5. Rhythm annotations (a) were accurately reproduced in over 90% of cases. Discrepancies were due primarily to inconsistencies in the original database. The therapy variables showed the largest error rates in mode (15%), impedance (12%), and energy (14%) annotations (b). Defibrillator generated data for these variables differed from estimates by the reviewers taken directly from defibrillator screen. Time variable (c) demonstrated a large number of unknown values. For those values that were present, the correlation of time values between the original database and the values recreated by the algorithm was over 90%. The missing values accounted for a large portion of values related to ECG start, first compression time, VF onset time, and time of return of spontaneous circulation. These are areas which require a judgment by the reviewer and therefore have a subjective component or may be obscured by CPR artifact or difficulty in ascertaining whether a pulse or blood pressure was present due to lack of documentation. The errors are divided between flaws in the algorithm and inconsistencies in the manual annotations.

In the following a detailed discussion is given on the various types of errors which occur.

### 4.1. Evaluation of the Replication of the Time Variables

The time variable results are shown in Figure 5 (for additional detail see Table 6).

For ECG start there are four errors. Three of these correspond to deviations of 4, 66, and 69 seconds and might be explained by special circumstances in the operation of the AED (see Appendix B.3 for details). One error corresponds to a deviation of more than 4 hours which we do not have an explanation for. Two of these deviations seem to propagate and might very well be the cause of corresponding deviations and reported errors for time of shock and time of first and last compression. Missing values were noted in 17 cases due to lack of a recorded value in the manual database. The automatic procedure used the date stamp found in the defibrillators files composing the CPR process data and therefore was always available.

For the time of shock (shktm), there are two errors propagated from deviations in ECG start time.

For the time of first compression (fctm) there are 50 deviations categorized as missing. In six of these cases the manual registration has provided a time for start of chest compressions corresponding to ECG start time. The algorithm has been designed to interpret this situation as “ongoing chest compressions” at the start of recording (see Appendix B.4 for details). One case is propagated from the large deviation in ECG start time (ectm). In the remaining 43 cases the algorithm produces the same missing codes as were used in the manual registration.

For the time of last compression (lctm) there is one error corresponding to a deviation of two seconds which we do not consider to be unacceptable. There are 31 cases coded as “missing.” There is one “missing” case where fc is given a time and lc is coded as unknown in the manual registration. The algorithm was designed to handle several variations of special coding of lc/fc and this is the only case not handled (see Appendix B.5 for details). One case corresponds to the deviation propagated from ecg start time. In the remaining 29 cases the algorithm produces the same missing codes as were used in the manual registration.

For the time of VF onset (vfonsettm) there are five deviations considered as errors. Four of these errors are deviations in the range 21-22 seconds. In the manual registration vfonset is set at 11-12 seconds after start of shock. In these cases VF is also annotated as reappearing 30 seconds after shock (r30). The algorithm makes the determination of VF onset at r30 and produces this offset in time compared to the manually derived reading by the reviewer. This discrepancy between the manual review time and the automatically derived algorithmic time appears to be explained by inaccuracies in determining the time point for the end of the shock. When the manual review noted the time at 10 seconds to be asystole and also recorded the vfonset to be in the 11–15-second range, the algorithm would define VF onset at the next rhythm time check at r30. (See Appendix B.6 for details.) There is one case where the deviation is one hour and the manual registration obviously is wrong as the time given precedes the start of episode. There are also six cases considered as “missing” where the manual registration has provided a proper time which the algorithm has interpreted as persistent VF. In all these cases the manual rhythm annotations prior to and after shock (rhyb4, r10, r30, and r60) indicate VF. The algorithm is designed to recognize these cases as persistent VF (in 38 cases the algorithm and manual registration coincide). There are two cases considered as missing where the manual interpretation indicates persistent VF while the algorithm has yielded a proper time. In the first case the manual rhythm annotations at 10 and 30 seconds after shock indicate asystole and VF, respectively (r10 = 1 and r30 = 2). This is interpreted by the algorithm as VF reappearing at 30 seconds. In the second case shocks 2 and 3 are not registered. The study is designed on the assumption that the episode registrations are complete. In this case, the rhythm annotations will not be correct as two shock registrations are missing. There is one case where the manual interpretation has used the code for persistent VF while the algorithm has used the “missing” code 88 : 88 : 88. In this case the preshock rhythm is VF and the rhythm at ten seconds is “unknown.” The algorithm has not been designed to recognize this as persistent VF (in 43 cases the algorithm and manual registration coincide). So in 81 out of the 90 cases categorized as missing, the algorithm produced the same missing codes as were used in the manual registration.

For time of ROSC (rsctm) there are 15 correct registrations and 125 considered as missing. The only differences are discrepancies in the use of codes for “unknown” 99 : 99 : 99 and 88 :  88  : 88 which the algorithm is not able to distinguish and therefore uses the “unknown" code consistently.

### 4.2. Evaluation of the Replication of the Patient Response Variables

The patient response variables are coded as correct if the manual and automatically generated values are identical. Otherwise the automatically generated variable is considered wrong. The patient response variables are shown in Figure 5 (for additional detail see Table 7). For the initial rhythm (init_rhy) there are no errors.

For the preshock rhythm (rhyb4), there are four errors. In three of these cases the last rhythm registration prior to the previous shock deviates from what has been manually determined as the preshock rhythm. The algorithm determines rhyb4 from the last registration prior to the current shock (r120, vfonsettm, or rosconsettm). The fourth error corresponds to the case of two missing shock registrations which corrupts the generation of RORE.

There are five errors for the rhythm at 10 seconds after shock (r10). For four of these cases, the manual registration indicates a non-VF rhythm. At the same time, the manual registrations of the time for VF onset (vfonsettm) are 13–16 seconds after shock. This will be reflected in the generation of RORE and the algorithm will look for rhythm transitions in a time window of five seconds that are present ten seconds after end of shock (end of shock is set to three seconds after shock is delivered). Thus, the algorithm will find a rhythm transition to VF corresponding to the registered time of VF onset. The fifth error corresponds to the case of two missing shock registrations which corrupts the generation of RORE.

There are four errors for the registration of the rhythm at 30 seconds after shock (r30). All of these are cases where the algorithm indicates VF corresponding to a VF onset time set at 30–35 seconds after shock rather than the manually registered non-VF rhythm. The explanation for this is similar to the one given for r10 above.

For the rhythm at 60 seconds after shock (r60) there is one case corresponding to the problem with VF onset and one corresponding to the 2 missing shock registrations both described above. The third error corresponds to a case where the manual registration has provided an unknown rhythm. In RORE, the transition to unknown rhythm will occur at 60 seconds. In the case of transition to unknown rhythms around the time point under consideration the algorithm is designed to choose the last known rhythm as long as it is present within the time window. This special handling was designed to handle transitions to unknown rhythms generically but does not work well when the time of rhythm transitions are not accurately represented. For the rhythm at 120 seconds after shock (r120) there are six errors. There are two cases corresponding to the problem with VF onset and one corresponding to the 2 missing shock registrations both described above. In another case, the manual registration indicates a proper rhythm while the algorithm has determined the rhythm as “unknown.” Here the ECG recording ends at 111 seconds after shock. This is represented in RORE as unknown rhythm (UN) and the algorithm consequently sets the rhythm at 120 seconds as “unknown.” There is also one case where the time of ROSC onset is 100 seconds after shock and the manual registration at 120 seconds indicates a transition to “unknown rhythm.” In this case, the algorithm will indicate the last known proper rhythm as described above. There is also a case with mismatch between “undetermined” and “unknown” rhythm.

For the indication of whether VF occurs prior to the next shock (vfpr), there are 2 errors. In both cases there is no evidence of VF in the interval in question. In one of the cases the algorithm states that there is no VF, contradicting the manual registration of “yes.” In the other case, the algorithm indicates “unknown” in contrast to the manually registered “no” because the rhythm annotations indicate “unknown” or “indeterminate” rhythm.

For the indication of whether an organized rhythm occurs prior to the next shock (orgpr), there are 7 errors. One error corresponds to the last error described above where there is no evidence of organized rhythm in the interval in question. The algorithm indicates “unknown” in contrast to the manually registered “no” because the rhythm annotations indicate “unknown” or “indeterminate” rhythm. One of the other errors is due to the problem with the two missing shocks described above. In the five remaining cases, the rhythm annotations indicate an organized rhythm in the interval in question which the algorithm recognizes and determines “yes” in contrast to the manual registration of “no.”

For the indication of presence of ROSC (rosc) there are 13 errors. In eight cases, the ROSC time is “no” and the manual indication of ROSC is “yes.” The algorithm does not interpret this correctly as it bases its interpretation from RORE which does not carry information about any occurrence of ROSC. In the five remaining cases the ROSC time is “indeterminate” and the algorithm indicates “no” ROSC as there is no evidence of ROSC in RORE while the manual registration says “unknown.”

### 4.3. Evaluation of the Replication of the Therapy and Device Operation Variables

The results for the therapy and device operation variables are shown in Figure 5 (for additional detail see Table 8). The variables are considered correct if the manually registered and corresponding automatically generated variables are identical.

There are no errors for the variables indicating shock number (shkn), number of shock sequences (ssrecord), and number of shocks in sequence (shks).

For the variable indicating the mode of the AED at the time of shock (mode) there are 21 errors which all correspond to the algorithm providing “yes” rather than “no” for manual mode. These are a result of the manual reviewer incorrectly assuming the mode was for automatic mode when in fact the device was in manual mode as indicated by the defibrillator process files.

For the variable indicating if there was EMS CPR prior to shock or not there are three errors. For two of the cases both start and end times of compressions prior to shock are set to “unknown.” The algorithm consistently interprets this as CPR being present (reducing error rates in tuning phase). The third error is due to the problem with the four-hour mismatch in the ECG start time variable.

For the variable indicating the impedance at first shock (imp) there are 17 errors.

For the variable indicating the energy of the first shock there are 20 errors.

The algorithm registers the impedance and energy for each individual shock. For these variables (except cpr) the automatically derived information is read directly from the log data file and is therefore exact. It is therefore an error caused by the person performing the manual extraction estimating the value and is not due to errors in the reasoning process of the algorithm.

## 5. Discussion

We have presented a method for replicating the manually annotated variables in an EMS registry database. The method was developed according to the principles presented by Eftestøl et al. [8] and implemented in MATLAB. To our knowledge, this is the first time automated review has been performed on resuscitation data.

In addition, the times, impedances, and defibrillation energies were obtained directly from the device logs and were inherently more accurate than visual estimates by reviewers. One of the main objectives of this study was to verify how closely the automated review can approximate the original data when one has access to the true annotations. These annotations are also the key information components used in the construction of the response and therapy representations that is fundamental to this method. As we discussed in the methods section, we used these representations to identify the shocks and determine the pre- and postshock events for each of these.

For the time variables, deviations larger than 1 second were categorized as errors. 5 seconds seems more appropriate as 1 second is very restrictive and identifies differences that are too small to be clinically significant. We suggest using a 5-second threshold in future studies. In the test data, there were only two errors in the 2–5-second range.

In the evaluation of the results, we have used the terms correct and wrong, but it is important to consider that we are really considering deviations between the manual and automatic registrations. The detailed review of the deviations showed that some of these were caused by errors in the manual registrations and others by errors in the automatic registrations. For the time variables, determining the vfonset was one such example where the problem was identified to be associated with the fact that manual interpretation of persistent VF is done in a time interval after shock and that the endpoint of a shock is not clearly defined for manual interpretation which it has to be for automatic interpretation. Otherwise, it seems that the algorithm greatly improves the accuracy over the human reviewer and is much more accurate. The machine is accurate to the millisecond providing that it has been properly synchronized to a “GMT” time and that relative times are clearly accurate to a millisecond barring machine malfunction. Human reviewers can only be accurate to about 1 to 2 seconds as we have shown in this work. As for the patient response variables, errors in the evaluation 10 seconds after shock can largely be associated with the nonprecise definition of the end of shock time. There are also errors that are caused by inconsistencies in the manual annotations. Generally speaking, the manual interpretation might cause errors in the sense that two different variables express the same information, like, for example, last rhythm change in the postshock period which is the same as the preshock rhythm in the next preshock sequence. The automatic annotations base its interpretation on considering the rhythms between uniquely defined rhythm transition times and thus avoids these kinds of inconsistencies. As for the interpretation of ROSC the algorithm does not interpret this correctly as it bases its interpretation from RORE which does not carry information about any occurrence of ROSC. For this case, the rhythm interpretation should distinguish between pulseless and pulse giving organised rhythms. For the therapy and device operation variables, the errors will mostly reflect errors in the manual registration as the algorithm reads this information directly from the electronic log files which will be reliable as long as the correct information is read. We have shown that we can replicate the review process of a given EMS system fairly accurately. To further develop this system and to handle the errors in the algorithms one would need to adjust the manual review process so that the critical time points are more clearly defined, make a dedicated study, and compare automatic and manual registrations again to see if errors are reduced. In an implementation, the automatic registrations should be checked and overread by a clinician.

The comparison shown above (Figure 5) demonstrates the inherent variability of a human reviewer in interpreting the data from the arrest and indicates several areas in which improvement can be made using an automated reasoning algorithm. One clear necessity is that all of the definitions must be made explicit so that a rule can be applied that will in every case provide the same result. This would allow the coding of computer algorithms to follow these definitions. In several cases (i.e., if VF that recurs after a shock is “persistent” or “recurrent” may depend on the duration of the period of asystole occurring after the shock). This study also clearly demonstrates that computer logs and times should be used in all cases as being more reliable than estimates and error prone manual data entry by reviewers. In this study the mode of defibrillator operation was incorrectly inferred by the reviewers in a high proportion of cases when the defibrillator log was able to provide this information accurately from the digitized files. In addition, a large number of errors which are “propagated” because the initial time point is unknown or recorded incorrectly can be reduced by improvements in using the time stamps from the defibrillator logs directly. These are examples of systematic deviations that further work with automatic methods of analysis can readily be programmed to reduce and eliminate.

The next step in developing this system will be to develop algorithms for automated rhythm annotations and chest compression detections. Our approach to do that will be to extract rhythm segments from the ECG tracings and categorize them according to the annotations used in the registry. The start and end points for each of these segments can be determined by using the same representations constructed in the present study. For example, all ECG tracings of VF segments without CPR artifacts can be found by searching the combined representation for each patient episode for the string “VF.” For all matches the VF segments can then be extracted from the ECG tracing as specified by the interval start and end sample given in the representation. Furthermore, signal processing algorithms for discriminating between the different rhythm categories will be developed. The discriminative power of these algorithms can be evaluated against the categorized collection of labeled rhythm segments. Correspondingly, methods for detecting chest compressions will be developed, applying the same principles for collecting segments with ongoing compressions from the signals carrying information about the presence of chest compressions. In this way, an EMS specific annotator can be made. This rhythm detector will replicate annotations as verified by blinded review by experts. Once rhythm and chest compression annotations are automated, the same algorithms used in this study for the construction of the representations from the manually extracted data can be applied. To evaluate the system, the performance of the fully automated system can be compared to the performance of the semiautomated and “expert reviewed” system which can be regarded as the gold standard.

In the current study, the presence of chest compressions was evaluated based on the compression depth signal derived from the acceleration measurements from the chest compression puck placed on the victim's chest. In the case where such information is not available, one might consider using the impedance data to follow respirations and one of our group is looking at how reliable this might be and also into adding the ETCO2 to the detail when it is available. The use of impedance for determining presence of CPR has been found to be reliable in a study by Stecher et al. [14]. This will allow the automatic reading using impedance in machines from other manufacturers who do not have the “puck” and in instances where the puck is not used even though it is at the scene. The impedance can also be used to automatically determine the presence of ventilations [15] and circulation [16] which has been demonstrated by Risdal et al. A future RORE can be extended to include information about ventilations, circulation, and possibly drug administration.

One method to validate the automatic analysis of the rhythm states would be for three expert reviewers to independently review the rhythm transitions and classifications as performed by the algorithm and to indicate errors or disagreements. One might then classify an “error” as any indication of disagreement with the algorithm that is noted by at least 2 of the 3 reviewers. Consideration of when the automatic method is accurate enough to be used without expert “overreading” would be based on a low error rate, perhaps less than 1%.

Of course, there are variables in the registry database that require manual interpretation. For example, there are variables describing the cause of CPR interruptions related to each shock and other variables for describing use of medications. The registration of these variables will still require manual interpretation.

Once a fully automated system is considered to perform satisfactorily, it will not only increase the efficiency for data interpretation at the EMS system from which it has been developed. It will also be possible to apply the automated review system to data from other EMS systems, thus enabling efficient multicenter data analysis without the need for centralized data storage. The results generated from data in this fashion will in general be anonymous with respect to issues of patient confidentiality. This method does access the CPR process data directly in the format downloaded from the AED device, thus making it independent from the local database structure used by the EMS. Thus interfacing the system directly to the raw data should make it easier to adopt it at other EMS sites with different registry database structures. Software interfaces will have to be adapted to read clinical information and translate it into the appropriate format.

In the present study we only considered a population of patients restricted to those treated with Philips AED device. In the case that we want to study data from different EMS services, the algorithms should also be able to handle data from other types of AED devices. One of the important aspects of this work is that as long as we are able to construct the state sequence models, we can apply the algorithms. So as long as the primary information objects are available through interpretation of the CPR process data it will be possible to apply the methods described here, independent of the type of AED device. Another objective of the present study was to demonstrate the applicability of the resuscitation data representation scheme presented by Eftestøl et al. [8]. The scheme was developed without knowledge of the data review method used in the King County EMS system. As was stated in that study, “We have discussed a methodology for representing resuscitation data emphasizing that such a representation scheme should offer a flexible format for efficient analysis of a variety of resuscitation research objectives.” In the present study we have demonstrated that the representation scheme offers a flexible format for efficient analysis. By applying the method to a research objective for which it was not originally intended, the representations in the three domains have been shown to be robust as well.

## 6. Conclusion

We have demonstrated that it is possible to use the information present in the CPR process data recorded by the AED during resuscitation along with rhythm and chest compression annotations to automate the episode review. This automated review is based on representing the resuscitation episode with sufficient detail using a minimal but sufficient set of transitions between rhythm states and therapy states which can be combined into a representation of the resuscitation episode which communicates the necessary information in a brief and compact format. This method can be automated to allow the development of a large database of resuscitation data for use in clinical studies of care and therapy for the cardiac arrest patient.

## Figures and Tables

**Table 1:** The time variables with descriptive names, variable names, coding, and explanation of each variable.

	Variable in Access database	Possible values	Description
ECG start time	ecghr	(0–23, 99) 99 = unknown	The hour of actual start time of the ECG
ECG start time	ecgmn	(0–59, 99) 99 = unknown	The minute of the actual start time of the ECG
ECG start time	ecgsc	(0–59, 99) 99 = unknown	The second of the actual start time of the ECG
First compression time	fchr	(0–23, 88, 99) 88 = no CPR administered, 99 = unknown	Hour of first compression, prior to shock. *Record only if first compression on record is actually the first compression given *
First compression time	fcmn	(0–59, 88, 99) 88 = no CPR administered, 99 = unknown	Minute of first compression, prior to shock. *Record only if first compression on record is actually the first compression given *
First compression time	fcsc	(0–59, 88, 99) 88 = no CPR administered, 99 = unknown	Second of first compression, prior to shock. *Record only if first compression on record is actually the first compression given *
Last compression time	lchr	(0–23, 88, 99) 88 = no CPR administered, 99 = unknown	Hour of last compression before shock
Last compression time	lcmn	(0–59, 88, 99) 88 = no CPR administered, 99 = unknown	Minute of last compression before shock
Last compression time	lcsc	(0–59, 88, 99) 88 = no CPR administered, 99 = unknown	Second of last compression before shock
Shock time	shkhr	(0–23, 99) 99 = unknown	Actual hour of shock delivery
Shock time	shkmn	(0–59, 99) 99 = unknown	Actual minute of shock delivery
Shock time	shksc	(0–59, 99) 99 = unknown	Actual second of shock delivery
VF onset time	vfonsethr	(0–23, 99) 99 = no onset, patient remained in VF blank indicates no vf	Hour of VF onset, best estimate when underneath CPR artifact
VF onset time	vfonsetmm	(0–59, 99) 99 = no onset, patient remained in VF blank indicates no vf	Minute of VF onset, best estimate when underneath CPR artifact
VF onset time	vfonsetss	(0–59, 99) 99 = no onset, patient remained in VF blank indicates no vf	Second of VF onset, best estimate when underneath CPR artifact *Therefore a 99* : *99* : *99 indicates an unsuccessful defibrillation *
ROSC time	rschr	(0–23, 99) 99 = unknown	Actual hour of ROSC
ROSC time	rscmn	(0–59, 99) 99 = unknown	Actual minute of ROSC
ROSC time	rscsc	(0–59, 99) 99 = unknown	Actual second of ROSC

**Table 2:** The variables describing device operation and therapy with descriptive names, variable names, coding, and explanation of each variable.

	Variable in Access database	Possible values	Description
Shock number	shkn	≥0 and <21	Shock sequence number.
Number of shock sequences recorded	ssrecord	(>0 and <31) or 99 99 = unknown	This describes the number of electrical shocks delivered to the patient as recorded on the AED total shocks received by the patient.
Number of shocks in sequence	shks	1–31	The number of shocks without intervening CPR. For example, 1 = no stacked shocks while > 1 = stacked shocks. After 2005 no case should have stacked shock.
Mode	mode	1 = manual 2 = semiautomatic & advisory 9 = unknown	This is the mode of the AED at the time of the shock
EMS CPR prior to shock?	cpr	1 = yes 2 = no 9 = unknown	Description of if EMS CPR was administered to patient before shock was delivered.
Impedance at 1st shock	imp	Measured in Ohms	Impedence at time of first shock sequence (50–200, 999) 999 = unknown.
Energy of 1st shock	enrgy	Measured in Joules	Energy of first shock in sequence.

**Table 3:** The response variables with descriptive names, variable names, coding, and explanation of each variable.

	Variable in Access database	Possible values	Description
Initial rhythm	init_rhy	1 = asystole, 2 = VF, 3 = pulseless VT, 4 = organized, 5 = indeterminate (VF/organized), 6 = indeterminate (VF/asystole), 7 = indeterminate (brady), 8 = indeterminate, 9 = unknown	Description of what the initial rhythm recorded was as determined from the AED. Codes in parenthesis refer to indeterminate between the two stated rhythms. If initial rhythm NOT VF and patient subsequently fibrillates, record original VF onset time, for substudy of secondary VF.

Preshock rhythm	rhyb4	1 = asystole, 2 = vf, 3 = vt, 4 = org, 5 = indeterm-(vf/org), 6 = indeterm-(vf/asys), 7 = indeterm-brady, 8 = indeterm, 9 = unknown	Description of what the preshock rhythm recorded was. Codes in parenthesis refer to indeterminate between the two stated rhythms.

Rhythm 10 secs after the last shock	r10	1 = asystole, 2 = vf, 3 = vt, 4 = org, 5 = indeterm-(vf/org), 6 = indeterm-(vf/asys), 7 = indeterm-brady, 8 = indeterm, 9 = unknown	Description of what rhythm recorded 10 seconds after the shock was. Codes in parenthesis refer to indeterminate between the two stated rhythms. Use discretion to take the rhythm ±5 seconds from 10 seconds after shock.

Rhythm 30 secs after the last shock	r30	1 = asystole, 2 = vf, 3 = vt, 4 = org, 5 = indeterm-(vf/org), 6 = indeterm-(vf/asys), 7 = indeterm-brady, 8 = indeterm, 9 = unknown	Description of what rhythm recorded 30 seconds after the shock was. Codes in parenthesis refer to indeterminate between the two stated rhythms. Use discretion to take the rhythm ±5 seconds from 30 seconds after shock.

Rhythm 60 secs after the last shock	r60	1 = asystole, 2 = vf, 3 = vt, 4 = org, 5 = indeterm-(vf/org), 6 = indeterm-(vf/asys), 7 = indeterm-brady, 8 = indeterm, 9 = unknown	Description of what rhythm recorded 60 seconds after the shock was. Codes in parenthesis refer to indeterminate between the two stated rhythms Use discretion to take the rhythm ±5 seconds from 60 seconds after shock.

Rhythm 120 sec after the last shock	r120	1 = asystole, 2 = vf, 3 = vt, 4 = org, 5 = indeterm-(vf/org), 6 = indeterm-(vf/asys), 7 = indeterm-brady, 8 = indeterm, 9 = unknown	Description of what rhythm recorded 120 seconds after the shock was. Codes in parenthesis refer to indeterminate between the two stated rhythms. Use discretion to take the rhythm ±5 seconds from 120 seconds after shock.

VF prior to next shock?	vfpr	1 = yes, 2 = no, and 9 = unknown	Description of whether or not there was VF between shocks, or between the last shock and the end of this recording as determined from audio or the MRIF. Not to capture VF at any time after device turned off.

Organized rhythm prior to next shock?	orgpr	1 = yes, 2 = no, and 9 = unknown	Description of whether or not there was an organized rhythm between shocks, or between the last shock and the end of the recording. Best organized rhythm (wide/narrow, and rate) will be taken within 3 minutes of the final shock on recording.

ROSC	rosc	1 = yes, 2 = no, and 9 = unknown	Description of whether return of spontaneous circulation occurred.

**Table 4:** The primary information objects (PIO). These variables constitute the primary information objects (PIO) and are used to model the entire resuscitation episode. The therapy and response domains are described independently. For each domain the entire time span of the episode is described as a sequence of interchanging states. For each state change, the corresponding transition times are given which specify the times entering and leaving the state. The entering time of a state corresponds to the leaving time of the prior state unless it is the beginning of the episode. The leaving time of a state corresponds to the entering time for the next state unless it is the end of the episode.

Variable domain				
Transition time	Patient's response		Therapy	
	State	Code	State	Code
Seconds	Ventricular fibrillation	VF	Chest compressions	C
Seconds	Ventricular tachycardia	VT	Hands off interval	H
Seconds	Asystole	AS	Defibrillation	D
Seconds	Pulseless electrical activity	PE	Unknown	U
Seconds	Pulse generating rhythm	PR		
Seconds	Unknown	UN		

**Table 5:** The variables in the original database which will be automatically replicated. The table is organized in columns to highlight the type of information the variables provide: time events, patient's response, and device operation and therapy. Each variable is labeled according to it being a primary information object (PIO) or secondary information object (SIO). All the variables labeled as PIO can also be found in the table listing the PIO variables (Table 4). The remaining variables labeled as SIO can be automatically derived from the PIO variables (listed in table PIO) by designing proper reasoning algorithms.

Variable domain					
Time events		Patient's response		Device operation and therapy	
First compression time	PIO	Initial rhythm	SIO	Shock number	SIO
Last compression time	PIO	Preshock rhythm	SIO	Number of shock seq. recorded	SIO
VF onset time	PIO	Rhythm 10 sec after last shock	SIO	Number of shocks in sequence	SIO
ROSC time	PIO	Rhythm 30 sec after last shock	SIO	Mode	SIO
Shock time	PIO	Rhythm 60 sec after last shock	SIO	EMS CPR prior to shock?	SIO
		Rhythm 120 sec after last shock	SIO	Impedance at 1st shock	SIO
ECG start time	SIO	VF prior to next shock?	SIO	Energy of 1st shock	SIO
		Org. rhythm prior to next shock?	SIO		
		ROSC	SIO		

**Table 6:** Results for the comparison between manual and automatic recording of time event variables. The table counts the number of correct, wrong, and missing values. An automatic recording is considered correct if the deviation from the manual recording is less than or equal to one second. Otherwise the recording is considered wrong. A recording is considered missing if the code 66 : 66 : 66, 88 : 88 : 88, 99 : 99 : 99 is used for either of the recordings or the manual recording was changed to 00 : 00 : 00 or 12 : 00 : 00.

Time variables						
Time point	ECG start	Defib shock	First compression	Last compression	VF onset	ROSC pulse
Abbreviated	ecgtm	shktm	fctm	lctm	vfonsettm	rsctm
Correct	33	138	90	108	45	15
Wrong	4	2	0	1	5	0
Missing	17	0	50	31	90	125

**Table 7:** Results for the comparison between manual and automatic recording of patient response variables. The table counts the number of correct and wrong values. An automatic recording is considered correct if the automatic recording is identical to the manual recording. Otherwise the recording is considered wrong.

Response variables									
Variable name	Initial rhythm	Preshock rhythm	Rhythm 10 secs after the last shock	Rhythm 30 secs after the last shock	Rhythm 60 secs after the last shock	Rhythm 120 secs after the last shock	VF prior to next shock?	Organized rhythm prior to next shock	ROSC
Abbreviated	init_rhy	rhyb4	r10	r30	r60	r120	vfpr	orgpr	rosc
Correct	140	136	135	136	137	134	138	133	127
Wrong	0	4	5	4	3	6	2	7	3

**Table 8:** Results for the comparison between manual and automatic recording of device operation and therapy variables for the data set. The table counts the number of correct and wrong values. For all variables except imp and enrgy an automatic recording is considered correct if the automatic recording is identical to the manual recording. Otherwise the recording is considered wrong. For imp and enrgy the numeric deviations between the manual and automatic recordings are considered. A deviation of zero is considered correct, and larger deviations are counted as errors.

Therapy variables							
Variable name	Shock number	Number of shock sequences recorded	Number of shocks in sequence	Mode	EMS CPR prior to shock?	Impedance at 1st shock	Energy of 1st shock
Abbreviated	shkn	ssrecord	shks	mode	cpr	imp	enrgy
Correct	140	140	140	119	137	123	120
Wrong	0	0	0	21	3	17	20

## Appendices

### A. Supplement—Reasoning from the Representation Back to Create the Derived Data

#### A.1. Time Data Conversions

The initial time event retrieved from the defibrillator is the “powerCycleOn” which corresponds to the time the ECG recording is started (0 seconds). This date stamp gives the date and time (“yyyy:mm:dd:hr:mn:sc”) at the start of the recording and is used to determine “ecgtm.” The datestamp is converted to a serial date number (“SDN_time”) which gives the time in number of days from January 1, 0000, and this is the SDN_time for the start of the recording. This initial event is called “Start_SDN.” The time as recorded in the RORE for all subsequent events found in the representations can then be converted to the SDN_time by taking the time (in seconds) recorded in the RORE for that event and convert it to an SDN_time which is in “Days Since 0000” as follows: SDN_time = Start_SDN + (Event_Seconds/60/60/24). The second term converts the number of seconds elapsed to the number of days elapsed as coded in SDN_time. Thus, to reason backwards, in order to determine the shock times in SDN_time from the RORE times recorded in seconds, the representation is searched for the symbol used to identify a shock, “D.” The time interval in seconds that coincides with each match is then converted to the SDN format which provides the elapsed time in days from Start_SDN. When this elapsed time is added to the Start_SDN it gives the year, date, and time accurately as encoded in the SDN format. This format could be used by itself as a reliable and reproducible time variable. In this study the data in the original ACCESS database was in the hour:minute:second format. For this reason we transformed the SDN format to hr:mn:sc format so that the derived data could be compared with the original data directly.

#### A.2. Determining the Postshock Rhythms at 10, 30, 60, and 120 Seconds

The postshock rhythms along with the start, end time and duration of each rhythm are derived from the postshock interval as it is displayed in the episode representation. If the rhythm at 10 seconds after shock is to be determined, the postshock rhythm with the transition time closest to ten seconds bounded upwards to 15 seconds is allocated to the variable “r10.” The same procedure is repeated for the variables “r30,” “r60,” and “r120” (always looking 5 seconds beyond the time in question).

### B. Supplement to the Results Section

#### B.1. Systematic Deviations in the ECG Start Time

When it comes to deviations in ecgtm, the algorithm differs between systematic and nonsystematic deviation. In the result evaluation of the manual and the automatically generated variables these two types of deviations were handled differently. When the manual given time was 00:00:00 or 12:00:00, these are considered systematic and are compensated for in the evaluation of error so that a single early or initial deviation does not propagate to the subsequent time variables. These deviations are not considered as errors. For ecgtm the code “missing” is used. The nonsystematic deviations are not compensated for in the evaluation of the other variables and possible systematic components in the deviations might propagate and be counted as errors in the other time variables.

#### B.2. Use of Special Codes for “Missing” Information

In the original database several codes were used to indicate persistent VF recurring immediately after a defibrillation shock, “no CPR data available” due to failure to use the puck or other technical problem and “unknown” when no information could be recorded because of severe artifact, failure to record, and so forth.

#### B.3. Nonsystematic Deviations in the ECG Start Time

A possible explanation for these errors might be that defibrillator was turned on and the leads were not attached to the patient for about 1 minute in two of these cases and that the manual abstractor started the “ECG time” when the leads were hooked up, but the “auto” reading from the CPR process defibrillator data gives the time when the device was turned on. This is an error of process in the manual part because the reader interprets the start differently from the machine start.

#### B.4. Deviations Categorized as Missing for the Start of Compression Times

These are all cases where fctm is manually registered to be at the start of the ECG recording or previous to this. The algorithm sets the state of therapy to “unknown” in the period prior to the start of the ECG recording. The corresponding therapy representation will then start with the symbols U, C where the time for the transition to compressions corresponds to the ECG start time. When the algorithm interprets this symbol sequence, it determines fctm as unknown. This was a way of handling the cases were the manual registration of “fctm” was “unknown” (41 cases).

#### B.5. Deviations Categorized as Missing for the End of Compression Times

In RORE this is coded with the end of the compression sequence coinciding with the defibrillation. In some cases where both fctm and/or lctm are coded as “unknown,” the RORE will set fctm to correspond to the start of ECG recording and lctm to correspond to the time of defibrillation. This is unrealistic but was done so that the algorithm could be designed to recognize the case of both variables manually registered as “unknown.” This is the sole case where only lctm is registered as “unknown” so the algorithm was not designed to recognize this.

#### B.6. Deviations in VF Onset Time

The algorithm evaluates rhythms with reference to end of the shock which is set to 3 seconds after start of shock. In all these cases, the variable indicating the rhythm prior to shock (rhyb4) has been annotated as VF while the annotation for the rhythm at ten seconds after the shock given as non-VF. In RORE, the transition time to non-VF is set at ten seconds, and the algorithm interprets this as a VF that persists for less than 10 seconds after shock which is not considered when looking for vfonsettm. If it lasts longer it is considered persistent. This is a problem that arises because there is no accurate rhythm annotation immediately after shock. This error could be eliminated by using the shock time as a rhythm transition point. Essentially the rhythm would be considered “unknown' until the ECG voltage returned to baseline after the shock at which time a determination could be made.

### C. Detailed Explanation of the RORE in Figure 4

The time shown corresponds to 0 to 1057 seconds. The tracings shown in Figure 3 correspond only to the period from 180 to 330 seconds. The left column is the therapy representation, showing the therapeutic interventions. For the first 46 seconds there is no ECG, so this is represented by “UN” (unknown therapy). At 46 seconds recording of ECG starts and compressions are observed on the compression depth signal. At 180 seconds, the chest compressions are interrupted and a hands off interval follows (represented by an “H”) and lasts until a shock is given at 196 seconds (represented by a “D”). The remainder of the case shows similar sequences of hands off interval, chest compressions, and defibrillations until no further annotations are given at the end of the recording.

The patient response representation is shown in the middle. It represents the same time span, showing the cardiac rhythms presented by the ECG. As for the therapy representation, the rhythm for the first 46 seconds is unknown (“UN”). At 46 seconds, the initial rhythm was recorded as “VF.” The next observation was made at 208 seconds (ten seconds after shock) and asystole was recorded (“AS”). This continued until 258 seconds where an organized rhythm was observed (‘represented by “PE”). The remainder of the case shows similar transitions between rhythms, first to “VF” and then to “PE” again and finally ROSC at 469 seconds. The documentation of rhythms ends at 856 seconds.

The episode representation combines the therapy and response representations showing the interaction between therapy and response. From 46 to 190 seconds, compressions are provided during VF. Compressions are interrupted at 180 seconds, preparing for the shock given at 196 seconds. The effect of the shock is observed at 208 seconds. This is an effect of the extrapolation of the response representation from the spreadsheet information. The true transition time should be prior to this. Following this, the effect of the compressions is evident through transitions from asystole to organized rhythm and then to VF. Following this compressions are interrupted and a new shock is given resulting in an organized rhythm and finally ROSC before documentation ends.
